# Association Between Adalimumab Dosing Interval and Uveitis Recurrence in Patients with Ankylosing Spondylitis

**DOI:** 10.3390/biomedicines13092089

**Published:** 2025-08-27

**Authors:** Yeo-Jin Lee, Soo Min Ahn, Seokchan Hong, Ji Seon Oh, Chang-Keun Lee, Bin Yoo, Yong-Gil Kim

**Affiliations:** 1 Department of Rheumatology, Asan Medical Center, University of Ulsan College of Medicine, Songpa-gu, Seoul 05505, Republic of Korea; 2 Department of Biochemistry and Molecular Biology, Asan Medical Center, University of Ulsan College of Medicine, Songpa-gu, Seoul 05505, Republic of Korea; 3 Department of Information Medicine, Big Data Research Center, Asan Medical Center, Seoul 05505, Republic of Korea

**Keywords:** ankylosing spondylitis, uveitis, tumor necrosis factor-alpha inhibitors

## Abstract

**Background/Objectives**: Ankylosing spondylitis (AS) is a chronic inflammatory disease frequently associated with acute uveitis (AU). Adalimumab, a monoclonal tumor necrosis factor (TNF) inhibitor, is widely used to manage AS and AU. While biologic tapering is recommended in stable AS, its impact on uveitis recurrence remains unclear. **Methods**: This real-world retrospective cohort study analyzed 65 AS patients with a history of AU who received adalimumab for ≥6 months. Time-dependent covariates, including the Bath Ankylosing Spondylitis Disease Activity Index (BASDAI), erythrocyte sedimentation rate (ESR), and adalimumab dosing interval, were incorporated into a logistic regression model using generalized estimating equations to identify risk factors for uveitis recurrence. Uveitis recurrence rates were compared among different dosing intervals: every 2 weeks (q2w), every 3 weeks (q3w), and every 4 weeks or longer (q4w+). **Results**: Among the 65 patients, 27 (41.5%) experienced a total of 50 AU flares during adalimumab treatment. Recurrence rates increased with dosing interval extension (1.5% for q2w, 3.6% for q3w, and 4.0% for q4w+; *p* = 0.036). In adjusted logistic analysis, the odds ratio (OR) for AU recurrence was significantly higher for q3w (OR = 3.766) and q4w+ (OR = 4.916) compared to q2w. Other factors, including age, sex, ESR, and BASDAI, were not significantly associated with uveitis recurrence. **Conclusions**: Extended dosing intervals of adalimumab in AS patients with prior AU may be associated with a higher prevalence of uveitis recurrence, even when axial symptoms remain controlled. Therefore, adalimumab spacing strategies should be implemented with caution in these patients to minimize the risk of uveitis recurrence.

## 1. Introduction

Ankylosing spondylitis (AS) is a chronic inflammatory disease primarily affecting the axial skeleton. It is frequently accompanied by extra-articular manifestations, such as acute uveitis (AU), inflammatory bowel disease, and psoriasis [1]. Among these, uveitis is the most common extra-articular manifestation of AS, with a reported prevalence of approximately 30–40% [1,2].

Monoclonal tumor necrosis factor (TNF) inhibitors, commonly used in managing AS, have demonstrated significant efficacy in controlling uveitis. Among these, adalimumab is not only FDA-approved for non-infectious uveitis involving the posterior segment but has also been shown to effectively manage uveitis associated with AS [3,4,5]. Rudwaleit et al. reported a 58% reduction in AU flares in patients with a history of AU, decreasing from 68.4 attacks per 100 patient-years before treatment [4]. Similarly, in a study by van Denderen et al., adalimumab treatment resulted in a 72% reduction in AU attacks, decreasing from 68 attacks per 100 patient-years before treatment [3]. Additionally, Kwon et al. recently reported that adalimumab effectively reduced both the recurrence and incidence of AU compared to other biologic agents, including golimumab, infliximab, secukinumab, and etanercept [5].

While these biologics are effective in managing both axial symptoms and uveitis, recent European Alliance of Associations for Rheumatology (EULAR) recommendations propose considering the tapering of biologic disease-modifying antirheumatic drugs (bDMARDs) in patients achieving sustained remission, citing evidence that tapering often succeeds in maintaining disease control, unlike abrupt discontinuation [6,7]. Similarly, Korean guidelines align with this perspective, which recommend considering tapering in axial spondyloarthritis (axSpA) patients with stable, long-term remission [8]. However, the available data on the impact of tapering adalimumab (or TNF inhibitors) on uveitis recurrence in AS patients is scarce, and its effects remain unclear. A systematic review on TNF inhibitor optimization, precisely dose spacing in non-infectious uveitis, concluded that it is difficult to draw definitive conclusions regarding the effects of optimization on uveitis [9]. Additionally, most of the studies in the review focused on patients with Behçet’s uveitis, with only two studies involving AS patients treated with etanercept [9,10,11].

Therefore, this study aimed to investigate whether extending the dosing interval of adalimumab influences the recurrence of uveitis in AS patients with a history of AU who are on adalimumab therapy.

## 2. Materials and Methods

### 2.1. Study Design and Population

This real-world study retrospectively analyzed the data of patients aged 18 years or older who were diagnosed with AS and had a history of AU, treated with adalimumab ≥6 months at a single tertiary center in Seoul, South Korea. Adalimumab treatment was initiated between December 2007 and June 2022. The diagnosis of AS was made according to the Modified New York criteria [12]. In accordance with the Health Insurance Review and Assessment Service (HIRA) insurance coverage criteria, adalimumab was initiated in patients with a Bath Ankylosing Spondylitis Disease Activity Index (BASDAI) score of 4 or higher despite NSAID administration. When patients maintained sustained remission for at least 6 months, the interval between adalimumab doses was extended based on shared decision-making between the patients and their rheumatologists. Generally, the dosing interval was extended from every two weeks to every three weeks and, if remission persisted, further extended to every four weeks. In cases of uveitis recurrence or disease flare, the adalimumab dosing interval was reduced to regain disease control. This study was conducted in accordance with the Declaration of Helsinki and its subsequent amendments. The Institutional Review Board of Asan Medical Center approved this study (IRB No. 2024-1014). Due to this study’s retrospective nature, the requirement for informed consent was waived.

### 2.2. Data Collection

The following patient data were collected from electronic medical records: baseline characteristics at the time of adalimumab initiation—HLA-B27 positivity, age, sex, BASDAI score, smoking status, comorbidities such as hypertension (HTN) and diabetes mellitus (DM), and laboratory data including erythrocyte sedimentation rate (ESR) and C-reactive protein (CRP)—time from AS diagnosis to adalimumab initiation, duration of adalimumab use, and time from adalimumab initiation to uveitis recurrence. Former or current smokers were classified as smokers. ESR, CRP, and BASDAI were measured at each outpatient visit (monthly for the first 6 months and every 1 to 3 months thereafter). The frequency of outpatient visits was determined based on insurance regulations and shared decision-making between the patient and the physician, considering disease activity. In South Korea, insurance regulations mandate that patients receiving biological agents must attend outpatient clinic visits at least once per month during the first six months of treatment, followed by visits every three months thereafter. In clinical practice, patients with stable disease are typically scheduled for visits every three months, whereas those experiencing disease flares may have their visit frequency increased to every 1–2 months. The adalimumab dosing interval and uveitis recurrence were assessed at each outpatient visit. Dosing intervals were calculated based on the actual time between injections, allowing for the inclusion of temporary adjustments such as interval shortening during flare-ups and subsequent reversion to previous schedules. For analysis, the dosing interval and recurrence status from the previous visit to the current visit were evaluated to assess disease status at the current visit. Uveitis recurrence was defined as a documented diagnosis of active uveitis confirmed via slit-lamp examination, conducted by an ophthalmologist at our institution or an affiliated external eye clinic.

### 2.3. Statistical Analysis

Categorical variables were described as frequencies and percentages (n, %), while continuous variables were expressed as means (standard deviations [SDs]) or medians (interquartile ranges [IQRs]). Categorical variables between the two groups were compared using the Chi-squared test or Fisher’s exact test. Parametric and non-parametric data between the two groups were compared using the independent t-test and the Wilcoxon rank-sum test, respectively. A logistic regression model using generalized estimating equations (GEE) was employed to identify factors associated with uveitis recurrence incorporating time-dependent covariates. Uveitis recurrence at each visit was considered the primary outcome. GEE was used to account for the correlation of repeated measures—ESR, CRP, BASDAI, adalimumab dosing schedule, and uveitis recurrence at each visit—within individual patients, with robust standard errors applied to ensure valid inference under possible misspecification of the correlation structure. This approach allowed for robust estimation of regression coefficients while adjusting for intra-patient correlation. Covariates in the multivariable analysis were selected based on their statistical significance in the univariable analysis. The results were presented as odds ratios (ORs) and 95% confidence intervals (CIs). All statistical analyses were conducted using SAS (version 9.4; SAS Institute, Cary, NC, USA) or SPSS (version 21.0; IBM, Armonk, NY, USA). A *p*-value of <0.05 was considered statistically significant.

## 3. Results

### 3.1. Baseline Characteristics

During the study period, a total of 65 patients with AS who initiated adalimumab treatment and had a history of AU were included (Table 1). Patients were followed for a mean duration of 69.75 months (range, 6–198 months) during adalimumab treatment. Sixty patients (92.3%) were biologics-naïve, and three patients (4.6%) received concomitant csDMARDs during adalimumab treatment. The mean age of the patients was 41.2 ± 11.0 years, and 69.2% were male. HLA-B27 was positive in 98.5% of the patients. Comorbidities included HTN in 13.9% and DM in 4.6% of the patients. Over half of the patients had a current or past history of smoking at the time of adalimumab initiation. Among these patients, 50 AU flares were documented: a total of 27 patients experienced a recurrence of uveitis while on adalimumab, whereas 38 did not (Supplementary Figure S1). The first recurrence occurred after a median of 37.5 months (18.8–70.5) following the initiation of adalimumab treatment. There were no differences between the uveitis recurrence and the non-recurrence groups regarding age, sex, comorbidities, or BASDAI scores at the time of adalimumab initiation. However, CRP levels were higher in the uveitis recurrence group, and the duration of adalimumab treatment was significantly longer. Regarding the dosing interval, among the total of 1892 visits, 935 visits (49.4%) were administered adalimumab at a 2-week interval, 555 visits (29.3%) were administered at a 3-week interval, and 402 visits (21.2%) were administered at an interval of 4 weeks or longer.

### 3.2. Recurrence Rate of Uveitis According to Adalimumab Dosing Interval

We analyzed data on adalimumab dosing intervals, ESR, CRP, and uveitis recurrence at each visit for patients with AS. Based on dosing schedules of q2w (every 2 weeks), q3w (every 3 weeks), and q4w+ (every 4 weeks or longer), the overall incidence rates of uveitis recurrence were 1.5% (14/935 visits) for q2w, 3.6% (20/555 visits) for q3w, and 4.0% (16/402 visits) for q4w+ (Table 2A). Focusing on the 27 patients who experienced uveitis recurrence during maintenance therapy with adalimumab, we analyzed 921 visits. In this subgroup, the uveitis recurrence rates were 3.0% (14/465 visits) for q2w, 6.3% (20/315 visits) for q3w, and 8.7% (16/141 visits) for q4w+ (Table 2B). Chi-square trend analysis demonstrated a significant association between dosing intervals and uveitis recurrence both in the overall analysis of all patients treated with adalimumab (*p* = 0.036) and in the subgroup of patients with uveitis recurrence during adalimumab maintenance (*p* < 0.001).

### 3.3. Factors Associated with Uveitis Recurrence: Logistic Regression Model Using Generalized Estimating Equations

A logistic regression analysis using GEE was performed to confirm the impact of dosing intervals on uveitis recurrence. Initially, an unadjusted [crude] analysis was conducted on all 65 patients receiving adalimumab with a history of AU (Table 3). This analysis identified ESR, BASDAI, and adalimumab dosing interval as significant variables. Specifically, compared to the q2w dosing schedule, the OR for uveitis recurrence was 3.589 for q3w dosing and 5.162 for dosing intervals of q4w+. In the adjusted analysis, which included variables significant in the unadjusted model, the dosing interval remained the only significant factor. Compared to q2w dosing, the OR for uveitis recurrence increased significantly to 3.766 for q3w and 4.916 for q4w+. Age, sex, HTN, and smoking were not significantly associated with uveitis recurrence.

Next, an analysis was conducted on the 27 patients who experienced uveitis recurrence during adalimumab treatment (Table 4). In the unadjusted analysis, the adalimumab dosing interval was significantly associated with an increased OR for uveitis recurrence as dosing intervals lengthened, while BASDAI was marginally non-significant. In the adjusted analysis, the dosing interval remained the only significant variable. Compared to q2w dosing, the OR for uveitis recurrence was 3.217 for q3w dosing and 4.664 for q4w+.

## 4. Discussion

This retrospective cohort study investigated the relationship between adalimumab dosing intervals and uveitis recurrence. The findings suggest that an extended dosing interval is associated with a higher recurrence rate of uveitis. Specifically, compared to the standard dosing interval of every 2 weeks, the risk of recurrence was higher when the interval was extended to 3 weeks and further escalated with intervals of 4 weeks or longer. This study provides meaningful real-world data by evaluating dosing intervals and laboratory data at each visit, offering insight into the association between adalimumab spacing and uveitis recurrence. Importantly, considerable variability in diagnostic and therapeutic approaches for non-infectious uveitis has been reported in large cohort studies, highlighting the need for more targeted, disease-specific management strategies [13].

Studies investigating the recurrence rate of uveitis in patients with AS undergoing TNF inhibitor spacing are scarce. A previous study analyzing the safety and efficacy of low-dose etanercept compared with the standard dosage reported no significant difference in the incidence rate of uveitis between the two groups [11]. However, that study did not exclusively focus on uveitis, and its inclusion criteria did not restrict participants to those with a prior history of uveitis. Furthermore, etanercept has been shown to be less effective than other monoclonal TNF inhibitors in controlling uveitis [5,14], which may limit its applicability in assessing the impact of TNF inhibitor spacing on uveitis recurrence.

In our study, 41.5% (27 out of 65) of patients with a history of uveitis experienced a recurrence. This finding is consistent with a study by Kwon et al., which examined recurrence-free survival rates in patients with a history of uveitis across different TNF inhibitors [15]. Their study reported a recurrence-free survival rate for adalimumab of approximately 0.6 at 70 months, a follow-up period comparable to the 70 months observed in our study. However, in a study by Rudwaleit et al., among patients with a history of uveitis followed for up to 20 weeks, 8.4% of those receiving adalimumab experienced a recurrence [4]. Although this proportion is lower than that observed in our study, the recurrence rate, calculated as 28.72 flares per 100 patient-years, was higher than the 13.2 flares per 100 patient-years observed in our research. This discrepancy is likely due to differences in follow-up duration, as longer follow-up periods increase the likelihood of recurrence but also expand the denominator in incidence rate calculations, leading to a lower overall rate. Additional contributing factors may include racial differences in uveitis recurrence, variations in disease duration among included patients, and differences in the types of medications. A meta-analysis reported a pooled prevalence of AU of 25.8%, with rates around 30% in North America and Europe but closer to the low 20% range in Asia and Latin America [16]. Additionally, the prevalence of AU more than doubled in patients with a disease duration of over 20 years compared to those with less than 10 years [16].

In this study, adalimumab spacing was more strongly associated with uveitis recurrence than other known factors for non-infectious uveitis, including age, female sex, and smoking [17]. However, factors associated with AU in AS may differ from those with non-infectious uveitis in general. For instance, a study of AS patients who met the modified New York criteria identified peripheral arthritis as a risk factor, while ESR, CRP, HLA-B27, and disease duration were not found to be significant factors for uveitis incidence [18]. Another cross-sectional study involving 225 patients with spondyloarthritis (SpA) identified disease duration and a family history of SpA as risk factors [19]. A recent study analyzing the incidence of the first episode of AU in SpA patients reported a significant decrease in incidence during the biologics era compared to the pre-biologics era [20]. The study identified HLA-B27 positivity and a family history of uveitis as risk factors for AU, while a disease duration of over 5 years and the presence of psoriasis were associated with a reduced risk. In contrast, our study focused on risk factors for uveitis recurrence, which may differ from studies analyzing risk factors for the first episode. Additionally, some studies have focused on a broader group of SpA patients rather than just AS patients, which may lead to differences in findings compared to our study [19,20]. Furthermore, in our cohort, 98.5% of patients were HLA-B27 positive, making it difficult to evaluate any association between HLA-B27 status and uveitis recurrence. Notably, the prevalence of HLA-B27 positivity varies by ethnicity and geographic region [21,22], which may influence the generalizability of these findings.

Recent studies across autoimmune diseases have examined the feasibility and safety of tapering TNF inhibitors in patients with sustained remission. In rheumatoid arthritis, trials such as RETRO and STRASS show that dose reduction may increase relapse risk but does not necessarily lead to radiographic progression, especially in well-controlled patients [23,24]. Long-term data from the DRESS trial suggest that disease activity—rather than drug tapering itself—is the primary determinant of structural outcomes [25]. In Crohn’s disease, the STORI trial demonstrates a 44% relapse rate within one year of infliximab discontinuation. However, patients with favorable clinical profiles experience fewer relapses, indicating that tapering may be appropriate for select patient populations [26]. In axial spondyloarthritis, the EULAR guidelines state that tapering of biologic DMARDs may be considered in patients with stable disease. Building on this evolving body of evidence, we aim to investigate the potential effect of tapering on extra-articular manifestations—specifically uveitis, the most common among patients with AS who remain otherwise stable in axial symptoms. This study represents a preliminary effort to address this question and provides real-world evidence suggesting an association between adalimumab dose spacing and uveitis recurrence.

This study has several limitations. First, it was a single-center retrospective study, which may limit its generalizability as it includes only patients followed at a tertiary hospital. Additionally, the retrospective design precludes causal inference; although we observed a significant association between extended adalimumab dosing intervals and uveitis recurrence, a direct causal relationship cannot be established. In particular, decisions to extend adalimumab dosing intervals were more often based on axial disease activity than on the presence or risk of uveitis recurrence. Another inherent limitation of the retrospective design is the possibility for confounding via indication, as patients in remission were more likely to undergo adalimumab spacing. While we adjusted for time-dependent covariates using GEE, unmeasured clinical factors may have influenced treatment decisions. To strengthen causal inference, future studies should consider using methods such as marginal structural models or inverse probability weighting. Second, the absence of data on adalimumab trough levels and anti-drug antibodies limited our ability to explore potential mechanistic explanations for uveitis recurrence—particularly regarding extended dosing intervals. Without therapeutic drug monitoring (TDM), it remains unclear whether uveitis recurrence is related to subtherapeutic drug exposure, immunogenicity, or other pharmacokinetic factors. While previous studies show that low drug levels and the presence of anti-drug antibodies are associated with treatment failure in systemic autoimmune diseases and certain types of non-infectious uveitis [27,28,29], most of this evidence relates to idiopathic uveitis, Behçet’s disease, or Vogt–Koyanagi–Harada syndrome rather than AS-associated anterior uveitis. Moreover, a previous study on peripheral spondyloarthritis reported no significant association between adalimumab trough levels and treatment response after 24 weeks [30], suggesting potential disease-specific variability. This uncertainty is also reflected in current clinical guidelines. For example, recent guidelines for Crohn’s disease state that current evidence does not support proactive TDM over reactive strategies or standard care [31], with similar reservations noted in guidelines for ulcerative colitis [32]. In rheumatoid arthritis and axial spondyloarthritis, the role of TDM remains under investigation rather than established in clinical practice [7,33]. Nevertheless, the absence of TDM data in our study remains a key limitation, especially in evaluating the effects of adalimumab dose spacing. Future prospective studies incorporating TDM will be essential to determine whether pharmacokinetic factors mediate the relationship between extended dosing interval and uveitis recurrence in patients with AS. Third, the overall number of patients was relatively small. However, the topic itself is challenging to investigate through prospective trials, and we believe this study adds valuable real-world data to a relatively underexplored area. Importantly, our analysis incorporated 1892 individual visits using logistic regression with GEE, allowing us to reflect longitudinal changes in laboratory values, disease activity, and treatment intervals. Adalimumab dosing intervals were captured dynamically at each visit, allowing the inclusion of temporary adjustments such as interval shortening during flare-ups or subsequent reversion. This approach helps to mitigate the limitations of a small patient number and strengthens the robustness of our findings.

## 5. Conclusions

In conclusion, this study is the first to evaluate the association between adalimumab dosing intervals and uveitis recurrence in patients with ankylosing spondylitis. Our findings raise the possibility that extended dosing intervals may be associated with a higher likelihood of uveitis recurrence, even in patients with stable axial symptoms. These results highlight the importance of individualized treatment decisions, particularly for patients with a history of uveitis. While the retrospective design limits causal inference, this study provides valuable real-world evidence and underscores the need for prospective research to confirm these findings and guide optimal dosing strategies for TNF inhibitor therapy.

## Figures and Tables

**Table 1:** Baseline characteristics of patients with AS and a history of AU who are on adalimumab.

	Total (*N* = 65)	Uveitis Recurrence		*p*-Value
		Yes (*N* = 27)	No (*N* = 38)	
Age, years	41.2 ± 11.0	41.8 ± 12.0	40.7 ± 10.3	0.693
Male, n (%)	45 (69.2)	20 (74.1)	25 (65.8)	0.476
HLA-B27, n (%)	64 (98.5)	26 (96.3)	38 (100)	0.415
HTN, n (%)	9 (13.9)	4 (14.8)	5 (13.2)	>0.999
DM, n (%)	3 (4.6)	3 (11.1)	0 (0)	0.067
Ever smoker, n (%)	36 (55.4)	14 (51.85)	23 (60.5)	0.323
BASDAI	6.44 ± 1.31	6.68 ± 1.25	6.27 ± 1.35	0.223
ESR, mm/h	26.97 ± 25.88	30.22 ± 26.41	24.66 ± 25.60	0.205
CRP, mg/dL	1.36 ± 2.26	2.03 ± 2.99	0.93 ± 1.55	0.015
Duration of AS, mo.	49.05 ± 72.11	45.81 ± 77.59	51.34 ± 68.93	0.496
Duration of adalimumab, mo.	69.75 ± 46.46	86.04 ± 45.80	58.18 ± 43.91	0.011
Number of visits by adalimumab dosing interval, n (%)				
Every 2 weeks	935 (49.4)	465 (50.5)	470 (48.4)	
Every 3 weeks	555 (29.3)	315 (34.2)	240 (24.7)	
Every 4 weeks or longer	402 (21.2)	141 (15.3)	261 (26.9)	
Duration from initiation of adalimumab to recurrence of uveitis, mo.		37.5 (18.8–70.5)		

Values are mean ± standard deviation, median (interquartile range), or n (%). AS: ankylosing spondylitis, AU: acute uveitis, HTN: hypertension, DM: diabetes mellitus, BASDAI: Bath Ankylosing Spondylitis Disease Activity Index, ESR: erythrocyte sedimentation rate, CRP: C-reactive protein, mo.: month.

**Table 2:** Recurrence rates of uveitis by adalimumab dosing intervals in patients with AS and a history of AU who are on adalimumab. (**A**): Data from 65 patients with AS and a history of uveitis receiving adalimumab. (**B**): Data from 27 patients with AS and a history of uveitis, focusing on those who experienced uveitis recurrence during adalimumab treatment (p-value analyzing by Chi-square trend test).

A. All Patients (*n* = 651,892 Visits)			*p*-Value
Dosing Interval	No Recurrence, n (%)	Recurrence (+), n (%)	0.036
Every 2 weeks	921 (98.5)	14 (1.5)	
Every 3 weeks	535 (96.4)	20 (3.6)	
Every 4 weeks or longer	386 (96.0)	16 (4.0)	
B. Patients with recurrence during treatment (*n* = 27, 921 visits)			*p*-Value
Dosing Interval	No Recurrence, n (%)	Recurrence (+), n (%)	<0.001
Every 2 weeks	451 (97.0)	14 (3.0)	
Every 3 weeks	295 (93.7)	20 (6.3)	
Every 4 weeks or longer	125 (91.3)	16 (8.7)	

**Table 3:** Factors associated with AU recurrence in AS patients on adalimumab (*n* = 65).

	OR	95% CI		*p*-Value
		Lower	Upper	
Panel A: Crude model				
Age	1.017	0.990	1.045	0.230
Female	0.846	0.352	2.034	0.709
HTN	1.943	0.820	4.601	0.131
Smoker	0.577	0.275	1.211	0.146
Time-dependent covariate (ESR)	1.020	1.003	1.037	0.020
Time-dependent covariate (CRP)	1.208	0.893	1.633	0.221
Time-dependent covariate (BASDAI)	1.222	1.016	1.469	0.033
Time-dependent covariate (interval of adalimumab)				<0.001
Every 2 weeks	1 (ref)			
Every 3 weeks	3.589	1.719	7.493	0.001
Every 4 weeks or longer	5.162	2.821	9.445	<0.001
Panel B: Adjusted model				
Time-dependent covariate (ESR)	1.014	0.996	1.032	0.138
Time-dependent covariate (BASDAI)	1.145	0.953	1.376	0.149
Time-dependent covariate (interval of adalimumab)				<0.001
Every 2 weeks	1 (ref)			
Every 3 weeks	3.766	1.854	7.651	<0.001
Every 4 weeks or longer	4.916	2.724	8.872	<0.001

AS: ankylosing spondylitis, AU: acute uveitis, OR: odds ratio, 95% CI: 95% confidence interval, HTN: hypertension, BASDAI: Bath Ankylosing Spondylitis Disease Activity Index, ESR: erythrocyte sedimentation rate, CRP: C-reactive protein.

**Table 4:** Factors associated with AU recurrence in AS patients on adalimumab within patients with uveitis recurrence during adalimumab treatment (*n* = 27).

	OR	95% CI		*p*-Value
		Lower	Upper	
Panel A: Crude model				
Age	1.015	0.993	1.037	0.172
Female	0.996	0.503	1.972	0.991
HTN	1.733	0.974	3.085	0.062
Smoker	0.801	0.449	1.430	0.453
Time-dependent covariate (ESR)	1.013	0.994	1.033	0.182
Time-dependent covariate (CRP)	1.129	0.696	1.831	0.624
Time-dependent covariate (BASDAI)	1.190	1.000	1.417	0.050
Time-dependent covariate (interval of adalimumab)				<0.001
Every 2 weeks	1 (ref)			
Every 3 weeks	3.161	1.581	6.320	0.001
Every 4 weeks or longer	5.107	2.919	8.937	<0.001
Panel B: Adjusted model				
Time-dependent covariate (BASDAI)	1.130	0.959	1.332	0.146
Time-dependent covariate (interval of adalimumab)				<0.001
Every 2 weeks	1 (ref)			
Every 3 weeks	3.217	1.624	6.372	<0.001
Every 4 weeks or longer	4.664	2.656	8.190	<0.001

AS: ankylosing spondylitis, AU: acute uveitis, OR: odds ratio, 95% CI: 95% confidence interval, HTN: hypertension, BASDAI: Bath Ankylosing Spondylitis Disease Activity Index, ESR: erythrocyte sedimentation rate, CRP: C-reactive protein.

## Data Availability

The data supporting this study’s findings are available on request from the corresponding author, YG Kim. The data are not publicly available due to ethical review board restrictions, as it contains information that could compromise the privacy of research participants.

## Supplementary Materials

The following supporting information can be downloaded at: https://www.mdpi.com/article/10.3390/biomedicines13092089/s1, Figure S1: Timeline of adalimumab dosing intervals and uveitis recurrence events in patients with recurrent uveitis (*N* = 27). Each horizontal bar represents the dosing schedule for an individual patient over time, color-coded by interval: orange for every 2 weeks (2w), green for every 3 weeks (3w), and blue for every 4 weeks (4w). Black symbols (▲) indicate timepoints of uveitis recurrence.
