# Supplementary material for: Association Between Adalimumab Dosing Interval and Uveitis Recurrence in Patients with Ankylosing Spondylitis
Source: Biomedicines. 2025 Aug 27;13(9):2089. doi: 10.3390/biomedicines13092089 (PMC12467712; doi:10.3390/biomedicines13092089)
Supplement: Supplementary file 1 [file biomedicines-13-02089-s001.zip › biomedicines-3747038-supplementary.pdf]

Supplementary Figure S1. Timeline of adalimumab dosing intervals and uveitis recurrence events in patients with recurrent uveitis (N = 27)

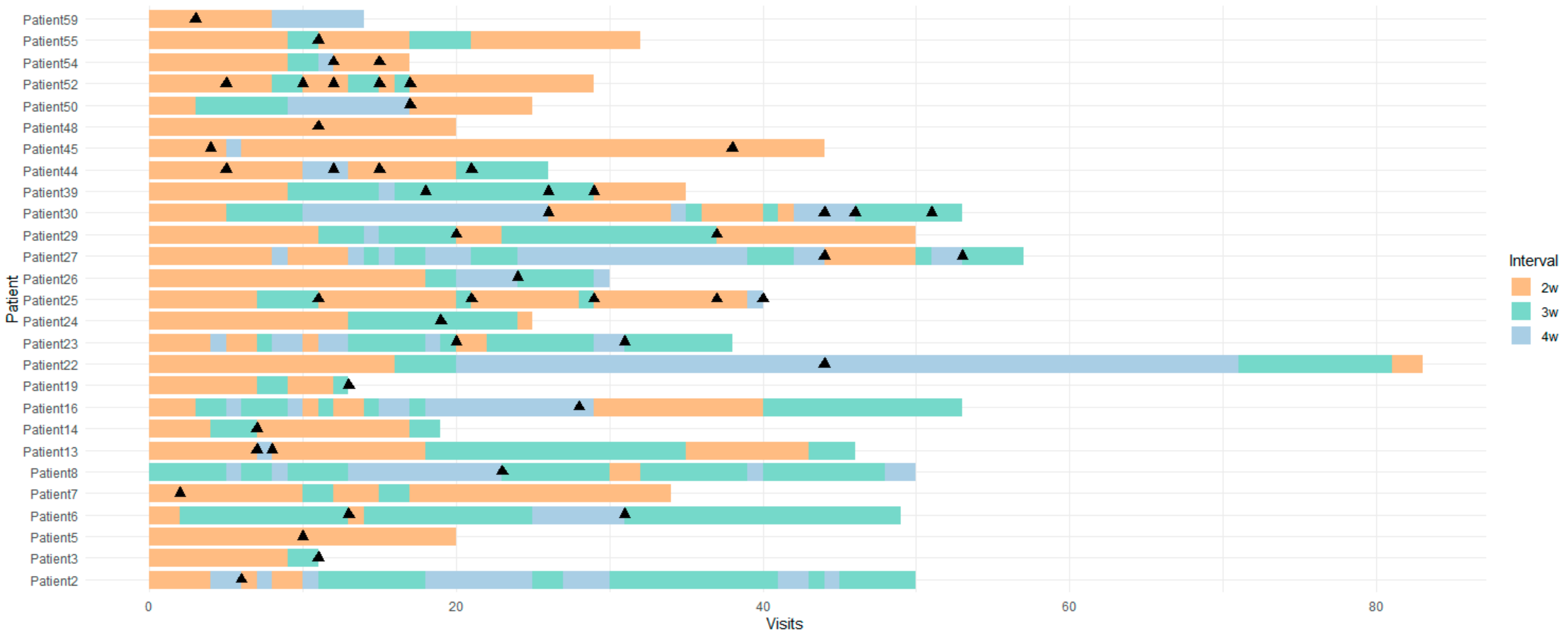

Each horizontal bar represents the dosing schedule for an individual patient over time, color-coded by interval: orange for every 2 weeks (2w), green for every 3 weeks (3w), and blue for every 4 weeks (4w). Black symbols (▲) indicate timepoints of uveitis recurrence.
